# Long Noncoding RNA Serve as a Potential Predictive Biomarker for Breast Cancer: A Meta-Analysis

**DOI:** 10.1155/2020/9045786

**Published:** 2020-05-10

**Authors:** Xuefeng Jiang, Guijuan Zhang, Jieyan Wu, Shujun Lin, Yusheng Liu, Yi Ma, Min Ma

**Affiliations:** ^1^College of Traditional Chinese Medicine of Jinan University, Guangzhou, China; ^2^The First Affiliated Hospital of Jinan University, Guangzhou, China; ^3^Institute of Biomedicine and Department of Cellular Biology, Jinan University, Guangzhou, China

## Abstract

**Purpose:**

The detection of long noncoding RNA (lncRNA) is a novel method for breast cancer diagnosis. The purpose of this meta-analysis was to evaluate the clinical significance of lncRNAs in identification of human breast cancer.

**Methods:**

Electronic databases, including PubMed (176), EMBASE (167), Cochrane Library (4), Web of Science (273), CNKI (41), VIP (18), and wanfang (21), were searched for relevant original articles. Diagnostic capacity of lncRNAs was assessed by pooled sensitivity and specificity, area under the summary receiver operating characteristic curve (AUC), diagnostic odds ratio (DOR), and subgroup and meta-regression analysis. Stata and Meta-Disc software were used to conduct the meta-analysis.

**Results:**

33 articles including 4500 cases were identified in our meta-analysis. lncRNAs sustained a high diagnostic efficacy; the pooled sensitivity, specificity, AUC, and DOR of lncRNAs in differentiating BC from controls were 0.74 (95% CI: 0.69-0.78), 0.78 (95% CI: 0.72-0.83), 0.82 (95% CI: 0.79-0.85), and 10.01 (95% CI: 7.13-14.06), respectively. The subgroup analysis showed that the diagnostic efficacy of lncRNAs in Asian populations was higher than that in Caucasians; lncRNAs in BC were lower than those in TNBC and were higher in plasma and serum specimens than in tissues. In addition, heterogeneity was clearly apparent but was not caused by the threshold effect.

**Conclusion:**

This meta-analysis suggested that lncRNAs might be promising biomarkers for identifying breast cancer, and its clinical application warrants further investigation.

## 1. Introduction

Breast cancer (BC) is the most common type of cancer in women worldwide. The proportion of BC in the incidence and mortality of female malignant tumors is increasing year by year [1]. The incidence of BC ranks first among female malignant tumors in 161 countries, and the mortality of BC ranks first among 98 countries [2]. Meanwhile, according to the 2013 Global Burden of Disease study, BC represented 24.2% of all cancer cases and 15% of all cancer-related deaths among females [3]. In 2018, the American cancer center estimated that new cases of ductal and lobular carcinoma in situ (CIS) breast cancer in the United States would be about 266,120 and 63,960, respectively. And it alone was anticipated to account for 30% of all new cancer diagnoses in women [4]. According to an annual report on status of cancer in China, 2017, about 279,000 new BC cases are reported in China each year, up more than 2% each year.

Histological evaluation of biopsy is the gold diagnosis standard for BC, which is invasive and fails to diagnose cancer at an early stage. Mammography X-ray imaging is widely used in BC screening and detection, but its application in radiation is limited [5]. Currently, several specific biomarkers, such as the carcinoembryonic antigen (CEA), carbohydrate antigen 15-3 (CA15-3), progesterone receptor (PR), and estrogen receptor (ER), have been employed extensively to diagnose BC in clinic [6, 7]. However, they are not sensitive and accurate enough to predict the prognosis of BC. Therefore, highly effective diagnostic and biomarkers are needed for early detection of BC to provide precise and personalized treatment for patients.

Recently, genome-wide transcriptome studies have confirmed the existence of a large number of long noncoding RNAs (lncRNAs) in the organism, which in the past were dismissed as simply transcriptional “noise” [8]. lncRNAs are non-protein-coding RNA molecules with a sequence longer than 200 nucleotides [9]. Mounting evidence indicated that lncRNAs are an important layer of the genome regulatory network and work via diverse mechanisms in a series of biological processes, including chromatin modification, transcriptional regulation, and posttranscriptional regulation [10]. lncRNAs play important roles in BC biological processes, such as increasing cell proliferation, migration, and invasion abilities, as well as epithelial-to-mesenchymal transition [11, 12]. One of the most studied lncRNAs, MALAT1 (Metastasis-Associated Lung Adenocarcinoma Transcript 1) promotes tumor growth by regulating cell cycle. Downregulation of MALAT1 in vitro in cell lines of different cancer types leads to reduced cell proliferation by cell cycle arrest in G2/M phase and to cell apoptosis. This ultimately leads to a decrease in the ability of cells to invade and migrate [13]. The lncRNA-HOTAIR (Hox transcript antisense intergenic RNA) is upregulated in primary breast tumors and metastases, and its overexpression is associated with enhanced BC metastasis [14]. Multiple tumor-related lncRNAs have been found in cell lines, tissues, and body fluid of cancer patients. Therefore, these molecules are considered as potential molecular biomarker for cancer diagnosis, prognosis prediction, and therapeutic targets [15, 16].

The diagnostic efficacy of lncRNAs in BC has been proved by many recent studies. However, which kind of lncRNA has higher diagnostic efficacy or lncRNA in which kind of sample might be more appropriate for BC diagnosis is inconsistent in different studies. For instance, the sensitivity and specificity of RP11-445H22.4 for BC were 92 and 74%, respectively [17], while those of MALAT1 were 71% and 75%, respectively [18]. Thus, we conducted a meta-analysis to evaluate the diagnostic efficacy of lncRNAs in identification of BC, which was intended to provide valid evidence for further studies about lncRNA.

## 2. Materials and Methods

### 2.1. Literature Search Strategy

This review was registered in the PROSPERO International Prospective Register of Systematic Reviews and the registration number was CRD42019139914. The preferred reporting items for systematic reviews and meta-analyses (PRISMA) was followed [19]. Two researchers searched the electronic databases (PubMed, EMBASE, Cochrane Library, Web of Science, Chinese National Knowledge Infrastructure Database (CNKI), VIP, and wanfang) from the start of each database up to June 1, 2019. The search strategy used both MeSH terms and free textwords to increase the sensitivity of the search. The following search terms were included: “breast cancer”, “lncRNA”, and “diagnosis”. We searched PubMed using the following strategy: (“Breast Neoplasms” [Mesh] OR “Breast Neoplasm” OR “Neoplasm, Breast” OR “Breast Tumors” OR “Breast Tumor” OR “Tumor, Breast” OR “Tumors, Breast” OR “Neoplasms, Breast” OR “Breast Cancer” OR “Cancer, Breast” OR “Mammary Cancer” OR “Cancer, Mammary” OR “Cancers, Mammary” OR “Mammary Cancers” OR “Malignant Neoplasm of Breast” OR “Breast Malignant Neoplasm” OR “Breast Malignant Neoplasms” OR “Malignant Tumor of Breast” OR “Breast Malignant Tumor” OR “Breast Malignant Tumors” OR “Cancer of Breast” OR “Cancer of the Breast” OR “Mammary Carcinoma, Human” OR “Carcinoma, Human Mammary” OR “Carcinomas, Human Mammary” OR “Human Mammary Carcinomas” OR “Mammary Carcinomas, Human” OR “Human Mammary Carcinoma” OR “Mammary Neoplasms, Human” OR “Human Mammary Neoplasm” OR “Human Mammary Neoplasms” OR “Neoplasm, Human Mammary” OR “Neoplasms, Human Mammary” OR “Mammary Neoplasm, Human” OR “Breast Carcinoma” OR “Breast Carcinomas” OR “Carcinoma, Breast” OR “Carcinomas, Breast”) AND (“RNA, Long Noncoding” [Mesh] OR “Noncoding RNA, Long” OR “lncRNA” OR “Long ncRNA” OR “ncRNA, Long” OR “RNA, Long Non-Translated” OR “Long Non-Translated RNA” OR “Non-Translated RNA, Long” OR “RNA, Long Non Translated” OR “Long Non-Coding RNA” OR “Long Non Coding RNA” OR “Non-Coding RNA, Long” OR “RNA, Long Non-Coding” OR “Long Non-Protein-Coding RNA” OR “Long Non Protein Coding RNA” OR “Non-Protein-Coding RNA, Long” OR “RNA, Long Non-Protein-Coding” OR “Long Noncoding RNA” OR “RNA, Long Untranslated” OR “Long Untranslated RNA” OR “Untranslated RNA, Long” OR “Long ncRNAs” OR “ncRNAs, Long” OR “Long Intergenic Non-Protein Coding RNA” OR “Long Intergenic Non Protein Coding RNA” OR “LincRNAs” OR “LINC RNA”) AND (“Sensitivity and Specificity” [Mesh] OR “Specificity and Sensitivity” OR “Sensitivity” OR “Specificity” OR “Diagnose” OR “Diagnosis” OR “Diagnostic” OR “Predictive Value of Tests” [Mesh]). Additionally, the reference lists of all included articles were also consulted to locate additional references of interest.

### 2.2. Inclusion and Exclusion Criteria

The inclusion criteria were as follows: (1) patients were diagnosed with BC by histopathological examination; (2) studies evaluated the diagnosis capacity of lncRNA for BC; (3) trials were described as case-control study; (4) studies must provide enough data to evaluate diagnosis value of lncRNA in BC; and (5) all the publication languages were restricted to Chinese and English.

Trials were excluded if they did not meet the criteria above and included the following: (1) reviews, abstracts, letters, meeting, and case reports; (2) animal studies or in vitro studies; (3) studies in respect of survival or prognosis of BC; (4) the research could not find the outcome measurements; and (5) duplicate publications. Two independent researchers assessed and selected related studies to exclude the references which did not meet the inclusion criteria. If the content of a study arouse controversy, it would be discussed until agreement was reached.

### 2.3. Data Extraction and Quality Assessment

Two independent investigators performed the data extraction using a standardized data collection form. The following information was collected from each study: (1) basic information including first author, publication year, and country; (2) the information about patients: ethnicity, sample type, sample size, and specimen source; (3) the information of methods: detection methods, lncRNA type, expression status, and reference gene; and (4) the outcomes: cutoff value and diagnostic 4-fold contingency table (TP, FP, FN, and TN).

The Quality Assessment of Diagnostic Accuracy Studies 2 (QUADAS-2) was applied to assess the quality of eligible studies and the risk of bias. In QUADAS-2 tool, patients through the patient selection, index test, and reference standard as well as flow and timing were discussed by 4 key domains and each of which contained applicability concerns and risk of bias. A score of 1 was given to low risk of bias or high concern about applicability, 0 to high or unclear risk or low or unclear concern. And any discrepancies were resolved through discussion.

### 2.4. Statistical Analysis

The application value of lncRNA for diagnosing BC was assessed through calculating the corresponding 95% CI, sensitivity, specificity, positive likelihood ratio (PLR), negative likelihood ratio (NLR), and diagnostic OR (DOR). The property of diagnostic tests was evaluated by DOR and summary receiver operator characteristics (SROC), and the pooled AUC value was calculated. Heterogeneity among studies was estimated using Cochran's *Q* statistic and *I*^2^ tests. *P* < 0.05 or *I*^2^ > 50% was defined to have heterogeneity. We calculated sensitivity and specificity with their corresponding 95% CI using a bivariate model. The random effects model was used when there was significant statistical heterogeneity; otherwise, the fixed effects model was used [20]. It was necessary to make a subgroup analysis and sensitivity analysis to seek the source of the heterogeneity. Visual funnel plot and quantifiable Deeks' funnel plot were used to identify the potential publication bias. *P* < 0.05 was considered significant. All these analyses were conducted using Meta-Disc 1.4 (XI Cochrane Colloquium, Barcelona, Spain) and Stata 15.0 (Stata Corporation, College Station, TX, USA).

## 3. Results

### 3.1. Study Characteristics and Quality Assessment

**Figure 1:**
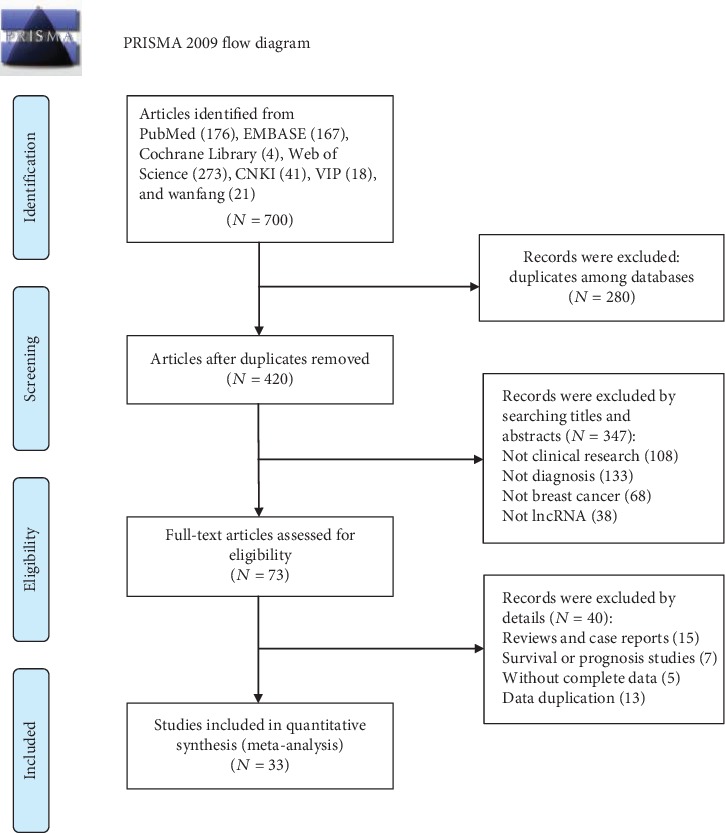
Flow diagram of the eligible studies.

**Figure 2:**
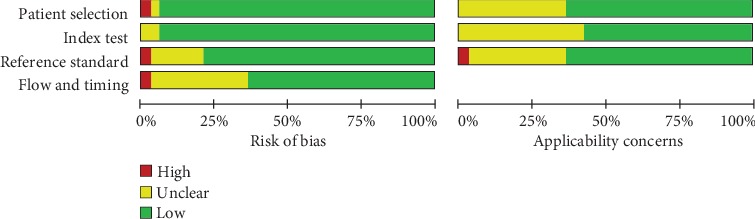
Study quality and bias assessment was conducted by QUADAS-2.

The detailed screening process was shown in Figure 1. A total of 700 articles were identified from the online database, and 480 articles were left after removing duplication. After screening the titles and abstracts and assessing the full articles, 33 eligible studies [17, 18, 21–51] were identified in the meta-analysis. These diagnostic studies involved 2425 patients and 2075 paired controls, and all participants were Asian and Caucasian. Most of the selected articles were published within the last 5 years. Regarding the specimen types, 9 studies examined tissue samples, 10 studies examined serum samples, 11 studies examined plasma samples, 2 studies examined plasma exosomal samples [46, 47], and 1 study examined urine samples [51]. Method of testing in all studies was real-time PCR, and lncRNAs were normalized by GAPDH, *β*-actin, and U6. These 33 selected cohorts were divided into 46 individual studies according to 30 lncRNAs (such as MALAT1, HOTAIR, and H19). The characteristics of the included studies were shown in Table 1. The Quality Assessment of Diagnostic Accuracy Studies 2 confirmed that all enrolled articles achieved a relatively high score equal or larger than 4, suggesting that these studies were reliable to be synthesized in the meta-analysis. The quality assessment was shown in Figure 2.

### 3.2. Diagnostic Efficacy of lncRNAs

**Figure 3:**
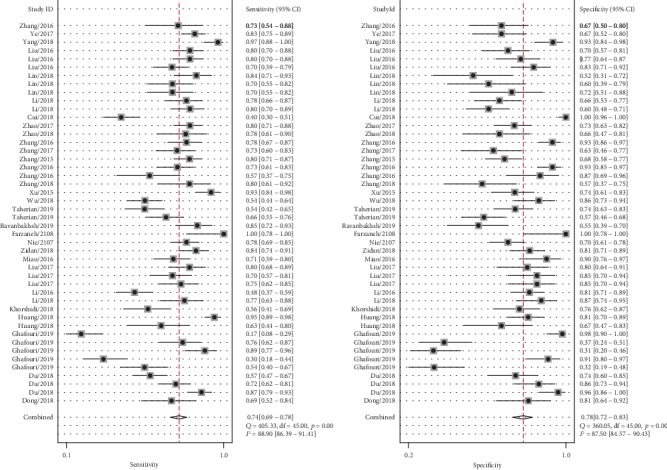
Forest plots of sensitivity and specificity of lncRNAs in the diagnosis of BC.

**Figure 4:**
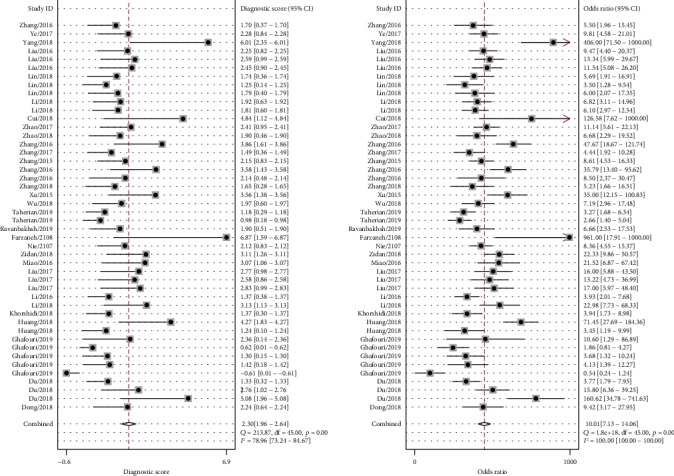
Forest plots of diagnostic odds ratio of lncRNAs in the diagnosis of BC.

**Figure 5:**
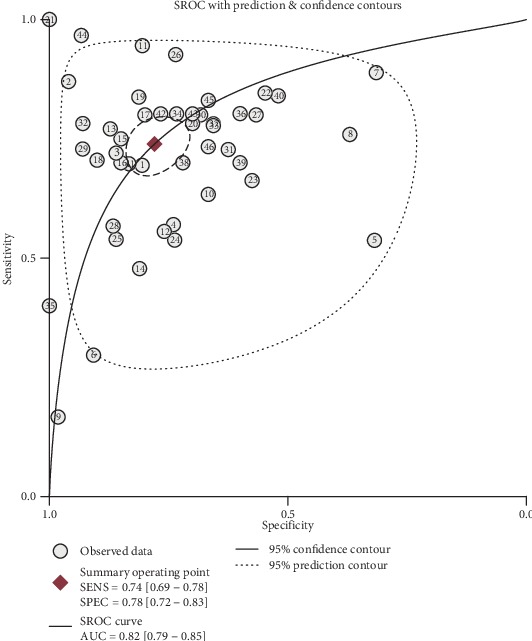
Summary receiver operating characteristic curve of lncRNAs for diagnostic value in BC.

**Figure 6:**
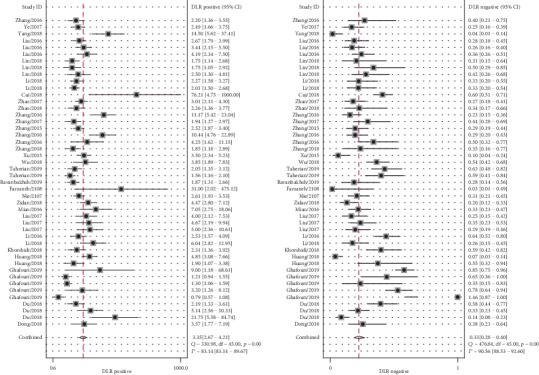
Forest plots of PLR and NLR of lncRNAs in the diagnosis of BC.

Significant heterogeneity was observed between studies for the high *I*^2^ values in sensitivity (88.90%, *P* < 0.001), specificity (87.50%, *P* < 0.001), PLR (83.14%, *P* < 0.001), and NLR (90.56%, *P* < 0.001). Therefore, the random effects model was adopted for further analysis. Forest plots of the results for the enrolled studies on the pooled sensitivity, specificity, and DOR were shown in Figures 3 and 4. The pooled sensitivity, specificity, and DOR of lncRNAs in differentiating BC from controls were 0.74 (95% CI: 0.69-0.78), 0.78 (95% CI: 0.72-0.83), and 10.01 (95% CI: 7.13-14.06), respectively. The summary receiver operator characteristic (SROC) curve was plotted and the AUC was calculated to be 0.82 (95% CI: 0.79-0.85) (Figure 5), indicating an overall high diagnostic accuracy as a diagnostic test. Moreover, the pooled positive likelihood ratio (PLR) and negative likelihood ratio (NLR) also were calculated 3.35 (95% CI: 2.67-4.21) and 0.33 (95% CI: 0.28-0.40) (Figure 6), respectively.

### 3.3. Analysis Diagnostic Threshold Effect

Threshold effect and nonthreshold effect are two important reasons for heterogeneity of diagnostic tests. In this study, Spearman's correlation coefficient of sensitivity and specificity was selected as the representative way of exploring the threshold effect. Since the corresponding Spearman correlation coefficient was 0.178 with a *P* value of 0.236 (*P* > 0.05), there was no heterogeneity from the threshold effect. In addition, the value of *I*^2^ > 50% indicated that there was heterogeneity of nonthreshold effect among these studies.

### 3.4. Subgroup and Meta-Regression Analysis

In order to illustrate the source of heterogeneity, we performed subgroup analyses based on ethnicity, pathologic type, specimen, dysregulated state, and type of lncRNA. The details were shown in Table 2. We found that the pooled sensitivity, specificity, and AUC of the studies were 0.76 (95% CI: 0.71-0.80), 0.80 (95% CI: 0.75-0.84), and 0.84 (95% CI: 0.81-0.87) for Asian versus 0.75 (95% CI: 0.71-0.78), 0.67 (95% CI: 0.49-0.80), and 0.72 (95% CI: 0.52-0.86) for Caucasian. When stratified by pathologic types, the diagnostic effect of lncRNAs in BC was lower than that in TNBC (AUC: 0.81 (95% CI: 0.77-0.84) versus 0.87 (95% CI: 0.84-0.90)). Subgroup analysis of specimens showed no significant difference in diagnostic accuracy between plasma and serum, with AUC of 0.86 (95% CI: 0.82-0.89) versus 0.86 (95% CI: 0.83-0.89), sensitivity of 0.76 (95% CI: 0.70-0.81) versus 0.78 (95% CI: 0.70-0.85), and specificity of 0.83 (95% CI: 0.76-0.88) versus 0.80 (95% CI: 0.70-0.87). However, both of these exhibited higher diagnostic accuracy than tissues, for which the pooled sensitivity, specificity, and AUC were 0.65 (95% CI: 0.52-0.76), 0.71 (95% CI: 0.57-0.83), and 0.73 (95% CI: 0.69-0.77), respectively. The diagnostic performance of lncRNA expression status suggested that upregulated lncRNAs (AUC: 0.84 (95% CI: 0.81-0.87)) were higher than downregulated lncRNAs (AUC: 0.70 (95% CI: 0.65-0.73)). In the meta-analyzed data based on types of lncRNA, lncRNA-MALAT1 sensitivity was 0.81 (95% CI: 0.71-0.88), specificity was 0.77 (95% CI: 0.68-0.84), and AUC was 0.85 (95% CI: 0.82-0.88), which were superior to lncRNA-HOTAIR and lncRNA-H19.

Meta-regression analysis indicated that ethnicity (*P* = 0.186), pathologic type (*P* = 0.428), specimen (*P* = 0.157), and type of lncRNA (*P* = 0.296) did not significantly affect the pooled results. However, lncRNA expression status was associated with study heterogeneity (*P* = 0.070).

### 3.5. Sensitivity Analysis and Publication Bias

**Figure 7:**
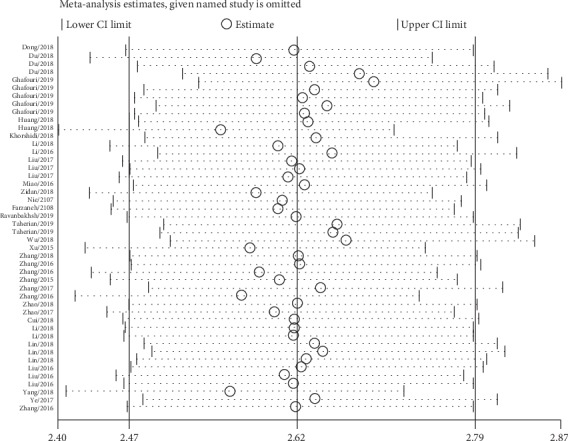
Sensitivity analysis of the overall pooled study.

Sensitivity analysis was used to test the stability of the overall effects and the change of heterogeneity by excluding a single study. None of the individual studies was out of the upper or lower CI limits, indicating that the selected studies were homogeneously distributed (Figure 7).

**Figure 8:**
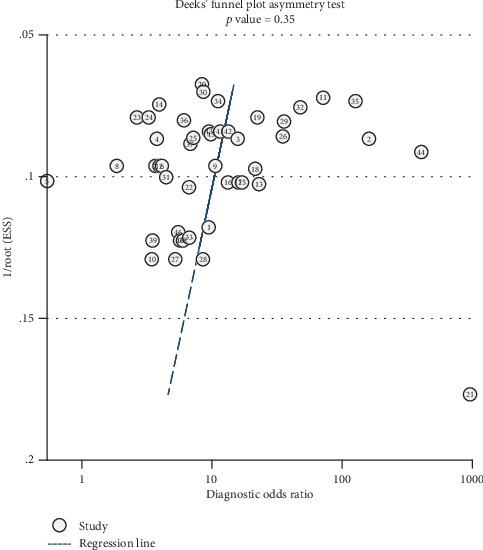
Deeks' funnel plots for the assessment of publication bias.

The publication bias among studies was assessed by Deeks' funnel plot asymmetry test. The symmetry of the funnel plot and a *P* value of 0.35 indicated that there was no significant publication bias in the diagnostic meta-analysis for lncRNAs (Figure 8).

### 3.6. Clinical Utility of lncRNAs in the Diagnosis of BC

**Figure 9:**
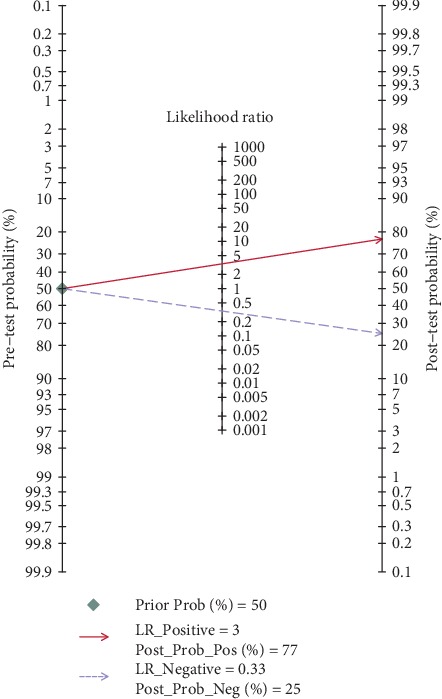
Fagan's nomogram of lncRNAs in the diagnosis of BC.

Fagan's nomogram was used to describe the diagnosis value of lncRNAs for BC (Figure 9). When 50% was selected as the pretest probability, the data indicated that, if the result of a diagnostic test was positive, the posttest probability that the individual suffered BC would be approximately 77% (red line). If the result was negative, the posttest probability that the participant was affected with BC would be approximately 25% (blue line), suggesting that lncRNAs were a promising indicator for the diagnosis of BC.

## 4. Discussion

BC is one of the top killers in women health. Given that the disease in early stages often appears with nonspecific symptoms, advanced or terminal diagnosis may occur by the time symptoms develop, with poor prognosis and poor treatment effect [52]. Mammography, ultrasonography, and magnetic resonance imaging (MRI) are routinely used for the detection of breast abnormalities. However, the sensitivity of mammography is moderate and can be affected by age and the density of the breast tissue. Breast ultrasonography has a high rate of false-negative results when used in BC screening, particularly in women with dense breast tissue. The high cost of breast MRI makes it inappropriate for use in screening for early BC [53]. Thus, there remains a considerable need for identification of novel biomarkers and better understanding of the molecular mechanisms underlying BC.

Increasing evidence suggested that lncRNAs are dysregulated in many different cancers, including BC [54, 55]. And the expression patterns of lncRNAs exhibit greater tissue specificity compared with protein-coding genes [56]. The functions of lncRNAs are complicated because they can function as oncogenic or tumor suppressor genes and positively/negatively regulate multiple targets by binding DNA, RNA, or proteins at multiple levels, including transcriptional and posttranscriptional levels [57, 58]. Recent studies have shown that lncRNAs are tumor-derived nucleic acids that can be released into the peripheral circulation and detected in the plasma and serum [59]. They can function as a competing endogenous RNA (ceRNA) that regulates other RNA transcripts by regulating specific miRNAs and the corresponding target genes [60]. For instance, lncRNA-HOTAIR is overexpressed in patients with BC, and its deregulation is correlated with enhanced BC metastasis [14]. lncRNA GAS5 inhibits the pathogenesis and progression and regulates the expression of PDCD4 of BC by acting as miR-21 “sponge” [61]. However, the function and overall clinical significance of the vast majority of lncRNAs in BC remain largely undetermined. Moreover, recent studies have demonstrated that lncRNAs exhibit greater expression restriction, and several lncRNAs have shown the potential as biomarkers for cancer diagnosis and prognosis [62]. Thus, it is definitely worthwhile to further investigate their potential roles and clinical utility in cancer progression.

lncRNAs have been proved to be a potential diagnostic biomarker for BC, although a meta-analysis about application value of lncRNAs on diagnosis of breast carcinoma had been published. Yu et al. evaluated 10 studies and showed that lncRNAs were highly sensitive (0.79 (95% CI: 0.70–0.85)) and specific (0.80 (95% CI: 0.73–0.85)) to diagnosis of breast carcinoma [63]. However, they included a limited number of studies and only assessed the pooled diagnostic efficacy of upregulated lncRNAs, but not the diagnostic performance of downregulated ones. According to the full text, Zhang et al. carried out a quantitative real-time PCR method to examine the expression levels of plasma H19 in 102 BC patients and 96 healthy controls, but only evaluated the diagnostic values of plasma H19 between 30 early-stage BC patients and 30 healthy controls [36]. Therefore, the number of samples included in Yu's meta-analysis may be biased. Furthermore, several studies about the diagnostic efficacy of lncRNAs have been published recently. So we conducted this meta-analysis to clarify the diagnostic effect of lncRNAs in BC.

The present meta-analysis for lncRNA expression profile in BC revealed that the pooled specificity and sensitivity were 0.74 (95% CI: 0.69-0.78) and 0.78 (95% CI: 0.72-0.83), which indicated its potential diagnostic capability. Diagnostic test performance was represented by DOR and area under SROC (AUC). The ideal SROC curve position for a perfect test is near the upper-left corner [64]. The DOR and AUC of lncRNAs were 10.01 (95% CI: 7.13-14.06) and 0.82 (95% CI: 0.79-0.85), respectively. Meanwhile, we also conducted a meta-analysis on the diagnostic efficacy of other serum biomarkers commonly used in clinic in the included literatures [17, 29, 34, 36, 39, 43, 49], such as CA15-3, CEA. The result showed that the pooled sensitivity, specificity, and AUC were 0.57 (95% CI: 0.42-0.70), 0.73 (95% CI: 0.68-0.78), and 0.73 (95% CI: 0.69-0.77) for CA15-3 and 0.61 (95% CI: 0.44-0.75), 0.51 (95% CI: 0.42-0.60), and 0.55 (95% CI: 0.51-0.59) for CEA. Our study found that lncRNA expression profile sustained a high diagnostic efficacy in identification of patients with BC from controls.

In our subgroup analysis by ethnicity, the results showed that the diagnostic efficacy of lncRNAs in Asian populations was higher than that in Caucasians (sensitivity: 0.76 (95% CI: 0.71-0.80) versus 0.75 (95% CI: 0.71-0.78); specificity: 0.80 (95% CI: 0.75-0.84) versus 0.67 (95% CI: 0.49-0.80); AUC: 0.84 (95% CI: 0.81-0.87) versus 0.72 (95% CI: 0.52-0.86)). For tumor types, the diagnostic effect of lncRNAs in BC was lower than that in TNBC (AUC: 0.81 (95% CI: 0.77-0.84) versus 0.87 (95% CI: 0.84-0.90)). The plasma and serum specimen sensitivity (0.76 (95% CI: 0.70-0.81) and 0.78 (95% CI: 0.70-0.85)), specificity (0.83 (95% CI: 0.76-0.88) and 0.80 (95% CI: 0.70-0.87)), and AUC (0.86 (95% CI: 0.82-0.89) and 0.86 (95% CI: 0.83-0.89)) were higher than tissue sensitivity (0.65 (95% CI: 0.52-0.76)), specificity (0.71 (95% CI: 0.57-0.83)), and AUC (0.73 (95% CI: 0.69-0.77)). The diagnostic performance of lncRNA expression status suggested that upregulated lncRNAs (AUC: 0.84 (95% CI: 0.81-0.87)) were better than downregulated lncRNAs (AUC: 0.70 (95% CI: 0.65-0.73)). lncRNA-MALAT1 sensitivity was 0.81 (95% CI: 0.71-0.88), specificity was 0.77 (95% CI: 0.68-0.84), and AUC was 0.85 (95% CI: 0.82-0.88), which were superior to lncRNA-HOTAIR and lncRNA-H19. Our findings may further help guide therapeutic strategy in the clinic.

Exploring the sources of heterogeneity is critical to a meta-analysis. Since heterogeneity was obviously revealed by test results, we attempted to explain its sources. Threshold effect is a primary cause of heterogeneity in test accuracy studies. The Spearman correlation coefficient was 0.178 (*P* = 0.236), which suggested that threshold effect was excluded from the factors causing heterogeneity in the current study. Sensitivity analysis was next used to test if the heterogeneity came from any individual study. Our results indicated that the selected studies were homogeneously distributed. Subgroup and meta-regression analyses were utilized for other factors causing heterogeneity. We validated five covariates, including ethnicity (*P* = 0.186), pathologic type (*P* = 0.428), specimen (*P* = 0.157), type of lncRNA (*P* = 0.296), and expression status (*P* = 0.070). The data suggested that lncRNA expression status was associated with study heterogeneity. Finally, Deeks' funnel plot showed that there was no significant publication bias in the diagnostic meta-analysis for lncRNA. Unfortunately, we failed to find other sources.

lncRNA appears to be a diagnostically valuable biomarker for BC. However, our meta-analysis has several limitations. First, most of the eligible studies were from China and Iran and included only Asian and Caucasian populations. Second, we selected 33 literatures, including 46 individual studies, which may lead to insufficient statistical sample capacity in statistics. A total of 30 lncRNAs were included in all studies, but some lncRNAs, such as SOX2OT, UCA1, USMycN, XIST, and Z38, were counted in only one study. Meanwhile, the subgroup analysis of the included studies was constrained by the limited number and size of available studies. Third, heterogeneity existed in the analysis and could not be explained by results of meta-regression and sensitivity analysis. Although the diagnosis values of lncRNAs were moderate, the performance of lncRNAs was not as satisfactory as expected based on the high accuracy criterion (NLR < 0.1, PLR > 10).

In conclusion, this meta-analysis comprehensively investigated the application of lncRNAs as a promising biomarker for diagnosis of breast carcinoma. Before the clinical use of lncRNAs as a diagnostic marker, more large-scale prospective studies are warranted to identify which lncRNAs have a better diagnostic role in BC.

## Figures and Tables

**Table 1:** Intervening characteristics of the included trials.

First author, year	Ethnicity	Pathologic type (E/C)	Sample size (E/C)	Specimen	lncRNA	State	Cutoff value	TP	FP	FN	TN	QUADAS-2
Dong, 2018	Asian	BC/ANCTs	36/36	Tissue	SNHG14	Up	Unclear	25	7	11	29	5
Du, 2018	Asian	TNBC/health	100/50	Plasma	ANRIL	Up	9.113	87	2	13	48	6
					SOX2OT	Up	9.113	72	7	28	43	
					ANRASSF1	Up	9.113	57	13	43	37	
Ghafouri, 2019	Caucasian	IDC/ANCTs	54/54	Tissue	FAS-AS	Down	Unclear	29	30	25	14	6
					TUG1	Down	Unclear	16	5	38	49	
					OIP5-AS1	Down	Unclear	48	37	6	17	
					NEAT1	Down	Unclear	41	34	13	20	
					HULC	Down	Unclear	9	1	45	53	
Huang, 2018	Asian	TNBC/health	30/30	Serum	MALAT1	Up	0.62	19	10	11	20	7
			128/77		MALAT1	Up	2.1345	121	15	7	62	
Khorshidi, 2018	Caucasian	IDC/ANCTs	54/54	Tissue	DSCAM-AS1	Up	Unclear	30	13	24	41	6
Li, 2018	Asian	TNBC/health	48/47	Serum	LINC00310	Up	1.402	37	6	11	41	4
Li, 2016	Asian	IDC/ANCTs	90/90	Tissue	GAS6-AS1	Down	5.21	43	17	47	73	6
Liu, 2017	Asian	TNBC/health	60/40	Plasma	ANRIL	Up	Unclear	45	6	15	34	5
					HIF1A-AS2	Up	Unclear	42	6	18	34	
					UCA1	Up	Unclear	48	8	12	32	
Miao, 2016	Asian	BC/BBD	78/40	Serum	MALAT1	Up	Unclear	55	4	23	36	4
Zidan, 2018	Caucasian	TNBC/health	80/80	Serum	MALAT1	Up	2.6	67	15	13	65	5
Nie, 2107	Asian	BC/ANCTs	110/110	Tissue	Z38	Up	2.86	86	33	24	77	5
Farzaneh, 2108	Caucasian	BC/ANCTs	15/15	Tissue	ARA	Up	Unclear	15	0	0	15	4
Ravanbakhsh, 2019	Caucasian	IDC/ANCTs	52/52	Tissue	USMycN	Up	17.5	44	19	8	23	5
Taherian, 2019	Caucasian	BC/ANCTs	80/80	Tissue	SNHG1	Up	Unclear	53	34	27	46	7
					SNHG5	Up	Unclear	43	21	37	59	
Wang, 2018	Asian	TNBC/health	68/64	Plasma	AWPPH	Up	Unclear					5
Wu, 2018	Asian	BC/health	102/50	Plasma	MALAT1	Up	Unclear	55	7	47	43	6
Xu, 2015	Asian	BC/health	68/68	Serum	RP11-445H22.4	Up	0.3	63	18	5	50	7
Yang, 2019	Asian	TNBC/health	26/38	Plasma	POU3F3	Up	Unclear					5
Zhang, 2018	Asian	BC/non-BC	30/30	Plasma	ROR	Up	1.205	24	13	6	17	7
Zhang, 2016	Asian	BC/health	30/30	Plasma	H19	Up	Unclear	17	4	13	26	5
Zhang, 2016	Asian	BC/health	70/86	Plasma	HOTAIR	Up	Unclear	51	6	19	80	4
Zhang, 2015	Asian	BC/health	100/104	Serum	HOTAIR	Up	0.3	80	33	20	71	6
Zhang, 2017	Asian	BC/health	66/40	Serum	MALAT1	Up	Unclear	48	15	18	25	4
Zhang, 2016	Asian	BC/health	88/100	Plasma	HOTAIR	Up	Unclear	61	7	17	93	6
Zhao, 2018	Asian	BC/health	36/32	Serum	XIST	Down	13.1	28	11	8	21	4
Zhao, 2017	Asian	BC/health	96/90	Plasma	ROR	Up	1.384	77	24	19	66	7
Cui, 2018	Asian	BC/health	90/94	Serum	LSINCT5	Up	1.433	36	0	54	94	7
Li, 2018	Asian	BC/health	76/80	Plasma	CRNDE	Up	Unclear	61	32	15	48	4
Li, 2018	Asian	BC/ANCTs	64/64	Tissue	MALAT1	Up	Unclear	50	22	14	42	4
Lin, 2018	Asian	BC/health	50/25	Plasma exosome	H19	Up	1.64	35	7	15	18	5
				Plasma	H19	Up	1.555	35	10	15	15	
Lin, 2018	Asian	BC/health	50/25	Plasma exosome	MALAT1	Up	0.743	42	12	8	13	5
Liu, 2016	Asian	BC/BBD	86/60	Serum	H19	Up	Unclear	60	10	26	50	4
					HOTAIR	Up	Unclear	69	14	17	46	
					MALAT1	Up	Unclear	69	18	17	42	
Yang, 2018	Asian	BC/BBD	60/60	Plasma	MALAT1	Up	Unclear	58	4	2	56	4
Ye, 2017	Asian	BC/health	124/48	Plasma	HIT	Down	Unclear	103	16	21	32	4
Zhang, 2016	Asian	BC/health	30/42	Urine	H19	Up	Unclear	22	14	8	28	4

Note: E/C: experimental group/control group; TP: true positive; FP: false positive; FN: false negative; TN: true negative; QUADAS-2: Quality Assessment of Diagnostic Accuracy Studies 2; BC: breast cancer; IDC: invasive ductal carcinoma; TNBC: triple-negative breast cancer; ANCTs: adjacent nontumor tissues; BBD: benign breast disease; MGF: mammary gland fibroma.

**Table 2:** Subgroup analysis of the diagnostic efficacy of lncRNA in breast cancer.

Parameter	No. of studies	No. of patients	AUC	Sensitivity	Specificity	Heterogeneity	Meta-regression (*P* value)
Ethnicity							0.186
Asian	35	4901	0.85 [0.81-0.87]	0.76 [0.71-0.80]	0.81 [0.75-0.85]	99%; 0.000	
Caucasian	11	1242	0.75 [0.71-0.78]	0.67 [0.49-0.80]	0.72 [0.52-0.86]	99%; 0.000	
Pathologic types							0.428
BC	36	4873	0.81 [0.77-0.84]	0.73 [0.66-0.78]	0.78 [0.70-0.84]	100%; 0.000	
TNBC	10	1270	0.87 [0.84-0.90]	0.78 [0.70-0.84]	0.83 [0.78-0.87]	73; 0.013	
Specimen							0.157
Tissue	14	1682	0.73 [0.69-0.77]	0.65 [0.52-0.76]	0.71 [0.57-0.83]	99%; 0.000	
Plasma	17	2560	0.86 [0.82-0.89]	0.76 [0.70-0.81]	0.85 [0.77-0.90]	98%; 0.000	
Serum	12	1679	0.86 [0.83-0.89]	0.78 [0.70-0.85]	0.80 [0.70-0.87]	98%; 0.000	
Dysregulated state							0.070
Upregulated	38	5193	0.84 [0.81-0.87]	0.76 [0.72-0.80]	0.81 [0.75-0.85]	99%; 0.000	
Downregulated	8	950	0.70 [0.65-0.73]	0.61 [0.40-0.78]	0.70 [0.44-0.87]	99%; 0.000	
lncRNA							0.296
MALAT1	10	1670	0.88 [0.85-0.90]	0.81 [0.71-0.88]	0.81 [0.68-0.90]	98%; 0.000	
H19	5	428	0.73 [0.69-0.77]	0.69 [0.63-0.75]	0.75 [0.65-0.83]	0; 0.242	
HOTAIR	4	684	0.82 [0.78-0.85]	0.78 [0.73-0.82]	0.85 [0.72-0.93]	89%; 0.000	

## Data Availability

The data used to support the findings of this study are included within the article.
